# News and Notes

**Published:** 1904-08

**Authors:** 


### *NEWS AND NOTES.*

JAPAN has 31,000 physicians and eight medical schools.

A NUMBER of cases of smallpox are reported from Niagara Falls.

THE two healthiest counties in the United States are Suffolk, L. I., and Berkshire, Mass.

THE Walter Reed Memorial Association has been incorporated under the laws of the District of Columbia.

THE City Council of Cleveland, Ohio, on May 31, passed an ordinance making it a misdemeanor to "use, give away, or sell a toy pistol or blank cartridge in this city on the Fourth of July."

THE Director of the Chicago Municipal Laboratory, Dr. J. F. Biehn, recently sent out a warning of danger of an epidemic of rabies. Muzzling of all dogs that appear in public is the remedy advocated by him.

THE National Association for the Study and Prevention of Tuberculosis was formally organized at Atlantic City June 6, with the following officers: President, Dr. E. L. Trudeau, Saranac Lake, N. Y.; secretary, Dr. E. Jacobs, Baltimore.

A BILL providing for the establishment of a bureau and a laboratory for the study of the criminal, pauper, and defective classes has been introduced into the New York State Legislature. It is based on a similar bill which has already been submitted to Congress. The work of the bureau would consist in the gathering of data of a sociological, pathological, or abnormal nature.

---

THREE of the most prominent members of the medical faculty of the University of Vienna have recently celebrated the anniversary of the day upon which they obtained their M.D. degree and were received into the profession. The anniversaries thus celebrated are the fiftieth, fortieth, and thirtieth—the fiftieth in the case of Weinlechner, professor of surgery; the fortieth in that of Toldt, professor of anatomy; and the thirtieth as regards Zuckerkanel, also professor of anatomy.

---

AMONG the resolutions adopted at the International Anti-tuberculosis Congress, held at Copenhagen during the last week in May, were those favoring the study of hygiene in schools and universities, uniform schedules for tuberculosis statistics, the appointment of a commission to consider the question of a predisposition to tuberculosis, and the enforcement of regulations against expectoration in places of public assemblage. Paris, says an exchange, was selected as the place for the next congress, which will be held in 1905.

---

AT the twenty-eighth annual meeting of the American Dermatological Association, which was held at the International Hotel, Niagara Falls, on June 2 and 3, 1904, the following officers were elected for the ensuing year: President, Dr. William T. Corlett, of Cleveland, Ohio; vice-president, Dr. Frank H. Montgomery, of Chicago; secretary and treasurer, Dr. Chas. J. White, of Boston; member of council, Dr. John A. Fordyce, of New York. It was decided to hold the next meeting of the association in New York City on December 28, 29 and 30, 1905.

IN San Antonio, Texas, a new force has been introduced in the warfare against the mosquito. Dr. Kohnke, the health officer of New Orleans, has met with great difficulties in his endeavors to "organize victory" against the baleful Anopheles, and this led him to conceive the idea of enlisting the aid of schoolboys in the work. He preached his new children's crusade at Laredo, San Antonio, Houston, and Galveston, and with the co-operation of the superintendent of the Board of Education of San Antonio he enrolled a number of the schoolchildren in his antimosquito army. It is proposed to carry on the fight on the same plan in New Orleans. It is certainly a happy thought to utilize the natural destructive tendencies of schoolboys for the extermination of these pestilent insects.

---

PROF. ELMER GATES, director of the Laboratory of Experimental Psychology at Washington, claims (*Practical Druggist*) to have produced the first absolute chemical vacuum known to science, and from which he has created rays which exhibit strange phenomena never mentioned as accompanying the Roentgen rays. The method of making the absolute vacuum was so simple and apparently effective that it is worthy of notice. He took a large, thick test-tube, made of the hardest potash glass, whose melting-point was at an extraordinarily high temperature. Into this he poured, while in a liquid form, a much softer glass, whose melting-point was at a comparatively low temperature. Allowing the liquid glass to cool gradually, it formed a solid mass with the tube. After attaching a suction piston to the mouth of the test-tube, the whole mass was slowly heated for about thirty hours. At the end of that time the softer glass became liquid again, while the tube still remained solid. By forcing the piston outward the greater part of the molteng lass was expelled. Enough was allowed to remain at the mouth of the tube to seal it by cooling in that position. Back of this stoppage there was left a space where there had never been the least quantity of gas, hence a complete and perfect vacuum.

---

AN exchange commenting on a medicolegal point says: "In trials for murder the point has sometimes been raised by the defense that death had not resulted from the wound inflicted by the accused, but from the subsequent surgical treatment, and that this should be considered in mitigation of the crime. We have the converse of this view in the case of the woman who stuck a knife into a man's heart and has been in jail awaiting the result of his injuries. By all the laws probably the man ought to have died. The wound was of a kind generally recognized as fatal. But the surgeons got hold of him, sewed up his heart, and in due time he recovered. Thereupon the woman was discharged from custody, and the man, in the exuberance of his delight, declares that she shall not be prosecuted. Some curious questions—moral, ethical, and legal—are raised by this extraordinary occurrence. How far was the degree of guilt or innocence which attached to the woman's act affected by the degree of perfection which surgery has attained? A few years ago a man so wounded would surely have died, and the person who inflicted the wound would have been convicted of manslaughter at least. Now the surgeons by a rare chance saved his life, and as nobody is the worse off no penalty is incurred. Are we to regard science as the handmaid of crime? And suppose the surgeons had failed, who would have been responsible?"

